# MAPK-RAP1A Signaling Enriched in Hepatocellular Carcinoma Is Associated With Favorable Tumor-Infiltrating Immune Cells and Clinical Prognosis

**DOI:** 10.3389/fonc.2021.649980

**Published:** 2021-06-10

**Authors:** Hailin Li, Guangyu Han, Xing Li, Bowen Li, Bo Wu, Hongyuan Jin, Lingli Wu, Wei Wang

**Affiliations:** ^1^ Department of General Surgery, Hongqi Hospital Affiliated to Mudanjiang Medical University, Mudanjiang, China; ^2^ Department of Oncology and Laparoscopy Surgery, The First Affiliated Hospital of Harbin Medical University, Harbin, China; ^3^ Department of General Surgery, The Fourth Affiliated Hospital of China Medical University, Shenyang, China; ^4^ Department of Cardiology, China–Japan Union Hospital of Jilin University, Changchun, China

**Keywords:** hepatocellular carcinoma, MAPK-RAP1A signaling, tumor-infiltrating immune cells, gamma delta T cell, prognosis

## Abstract

**Background:**

MAPK-RAP1A signaling, which is involved in cancer progression, remains to be defined. Upregulation of MAPK-RAP1A signaling accounts for most cancers that harbor high incident rate, such as non-small cell lung cancer (NSCLC) and pancreatic cancer, especially in hepatocellular carcinoma (HCC). MAPK-RAP1A signaling plays an important function as clinical diagnosis and prognostic value in cancers, and the role of MAPK-RAP1A signaling related with immune infiltration for HCC should be elucidated.

**Methods:**

Microarray data and patient cohort information from The Cancer Genome Atlas (TCGA; n = 425) and International Cancer Genome Consortium (ICGC; n = 405) were selected for validation. The Cox regression and least absolute shrinkage and selection operator (LASSO) were used to construct a clinical prognostic model in this analysis and validation study. We also tested the area under the curve (AUC) of the risk signature that could reflect the status of predictive power by determining model. MAPK-RAP1A signaling is also associated with tumor-infiltrating immune cells (TICs) as well as clinical parameters in HCC. The GSEA and CIBERSORT were used to calculate the proportion of TICs, which should be beneficial for the clinical characteristics (clinical stage, distant metastasis) and positively correlated with the survival of HCC patients.

**Results:**

HCC patients with enrichment of MAPK-RAP1A signaling were associated with clinical characteristics and favorable T cell gamma delta (V*δ* T cells), and STMN1, RAP1A, FLT3, HSPA8, ANGPT2, and PGF were used as candidate biomarkers for risk scores of HCC. To determine the molecular mechanism of this signature gene association, Gene Set Enrichment Analysis (GSEA) was proposed. Cytokine–cytokine receptor interaction, TGF-*β* signaling pathway, and Intestinal immune network for IgA production gene sets were closely related in MAPK-RAP1A gene sets. Thus, we established a novel prognostic prediction of HCC to deepen learning of MAPK-RAP1A signaling pathways.

**Conclusion:**

Our findings demonstrated that HCC patients with enrichment of MAPK-RAP1A signaling were associated with clinical characteristics and favorable T cell gamma delta (V*δ* T cells), which may be a novel prognostic prediction of HCC.

## Introduction

Hepatocellular carcinoma (HCC) is the sixth leading cancer and accounts for the second highest number of lethal malignant cancer globally (1). HCC accounts for most primary malignancy of liver cancers and is biologically heterogeneous. Despite treatment advances in surgery, liver transplantation, and radiofrequency ablation, the 5-year survival rate for HCC is still less due to heterogeneous tumor (2). At present, the development of sorafenib represented the main treatment for advanced liver cancer (3). However, a larger proportion of patients will have recurrence or metastasis (4). Thus, it is worth identify that distinct molecular genes and pathways, which accelerate molecular studies with the prognostic outcomes of HCC (5).

Among them, the MAPK and RAP1A are the mediator pathways in regulating cell biology of cancer cells (6, 7). MAPKs are best-characterized regulators of extracellular signals that are transduced to the nucleus; MAPKs include three important protein kinase families: extracellular signal-regulated protein kinases (ERKs), c-Jun NH2-terminal kinases (JNKs), and p38 family of kinases (8). Ras-associated protein-1 A (RAP1A), a small GTPase that belongs to the Ras-related protein family, was observed to regulate oncogenic Ras phenotype and Rho GTPase mediated actin cytoskeleton. RAP1A signaling is involved in cell proliferation, differentiation, and cell–cell junction in several cancer types (9–11). It was also reported that RAP1A regulates cancer through activating MAPK signaling in order to regulate motility and metastasis (12). The upregulation of RAP1A induced the MAPK signaling, which indicated that p38 may be identified as the counterparts of the RAP1A-dependent biological function (7, 12). Therefore, the clinical involved in the expression of MAPK-RAP1A signaling should be strongly considered.

The tumor microenvironment (TME), which contains stromal and immune cells, is thought to be a determinant factor in the advance of HCC (13, 14). Immune stroma plays a major role in the TME and development of tumor growth to the metastasis of HCC (15). Meanwhile, recent evidence indicates that immune cells may also play a role in cancer *via* ERK, another pathway of MAPK, thus probably essential for the development of therapeutic processes (16). Pancreatic cancer cells have been reported to be taken up by T lymphocytes to activate p38 MAPK *via* secreting exosomes, which ultimately causes immunosuppression (17). HCC-derived exosomes carried HMGB1 and activated TIM-1^+^ Breg cell; this delivery led to increase in angiogenesis by TLR and MAPK signaling pathways (18). To our knowledge, Ubc9 acts as a functional binding partner of ADAP and plays a selective role in TCR-mediated Rac1 activation *via* modulation of the membrane targeting of RAP1A and RapL (19). Therefore, the construction of MAPK-RAP1A signaling is known to involve the tumor microenvironment that can improve HCC prognosis.

In the present study, we constructed tumor-infiltrating immune cells (TICs) closely related to MAPK-RAP1A signaling through bioinformatics methods. Next, MAPK-RAP1A related signature genes significantly related to clinical features were detected to validate diagnosis and prognosis. Then we identified TICs by integrating MAPK-RAP1A related signature genes for HCC. We here aimed to provide potential marker for assessment of HCC clinical prognosis and playing a key role in TME.

## Materials and Methods

### HCC Patients

From January 2020 to December 2020, 20 human liver cancer tissue samples and corresponding para-carcinoma tissue samples were collected from patients with HCC in Hongqi Hospital Affiliated to Mudanjiang Medical University. The sample collection was approved by the research ethics committee of Hongqi Hospital Affiliated to Mudanjiang Medical University. Each patient provided informed consent to participate in the study. All collected samples were immediately frozen in liquid nitrogen until subsequent analysis.

### TCGA and ICGC Data Source

All eligible sequencing datasets, clinical characteristics, and follow-up information in The Cancer Genome Atlas (TCGA) (https://gdc.cancer.gov/about-data/publications/pancanatlas) and International Cancer Genome Consortium (ICGC) database (https://icgc.org/) were downloaded. Gene sets with HCC were used to perform KEGG analyses. The KEGG pathway in the TCGA database was used as the training set, and data in the ICGC data sets were used for the validation set. Meanwhile, MAPK-RAP1A related genes were selected using the R package “limma” from the pre-processed data with log2(x + 1) transformation, and the adjusted *P* value <0.05 was considered statistically significant.

### Construction of a Risk Signature Associated With MAPK-RAP1A Signaling

To screen the association between clinical stage and risk signature, multivariate cox regression models were used to assess the clinical characteristics of MAPK-RAP1A signaling in R language. The data in the TCGA database were used as the training sets, and data in the ICGC data sets were used for the validation sets to establish a nomogram. The HCC patients’ information identified the relationship between clinical stage and risk scores, and Wilcoxon rank test as the significance test. LASSO regression was used to set precision power gene combination to identify key modules (20). R language survival and survminer were applied for the survival analysis and time-dependent receiver operating characteristic (ROC) curve to assess the efficiency of the risk signature.

### Association of Hub Genes’ Expression With Tumor Purity and Tumor-Infiltrating Immune Cells

The ssGSEA and CIBERSORT tools were used to estimate tumor-infiltrating immune cells (TICs) for sample by R language (21, 22); the final estimate outcome was sum up to three kinds of scores: stromal score, immune score, and ESTIMATE Score, which correlated with the larger ratio of the immune infiltration. Moreover, the infiltration of T gamma delta showed a relatively lower risk signature in MAPK-RAP1A signaling. Next, we applied Tumor Immune Estimation Resource (TIMER) to integrate the results of those hub gene expression of HCC hub genes and both tumor purity, TICs. Interestingly, STMN1, RAP1A, FLT3, HSPA8, ANGPT2, and PGF were all positively associated with tumor purity. Furthermore, a relationship was observed between the six hub genes and infiltration of immune cells (23, 24). Combined with the above results, we suggested that the prognostic evaluation based on the high abundance of MAPK-RAP1A signaling assessed through ssGSEA or CIBERSORT analysis is reliable.

### Functional Enrichment Analysis

Gene set enrichment analysis (GSEA) was performed to investigate the MAPK-RAP1A signature gene potential mechanisms (21). The gene set “c2.cp.kegg.v6.2.symbols.gmt”, downloaded from the Molecular Signature Database (MSigDB, http://software.broadinstitute.org/gsea/msigdb/index.jsp), was selected as the reference gene set. GSEA was used to elucidate biological processes associated with the significant survival difference; R package “ClusterProfiler” was utilized for GSEA analysis of the hub genes between low expression group and high expression group in R with adjusted P <0.05 and |log_2_FC| >0.5 (25).

### Immunohistochemical Staining

Each resected specimen was fixed with 10% formalin, dehydrated, and embedded in paraffin. Paraffin sections were 3 μm thick and placed on glass slides. Firstly, the sections were deparaffined and incubated in an oven at 70°C for 1 h and 30 min. Then sections were carried out in xylene, and rehydration was carried out after gradient ethanol dehydration. Afterwards, the sections were incubated in 1× EDTA solution at low strength of microwave for 15 min to keep the endogenous peroxidase activity. Then, the sections were washed with PBS for three times and were then incubated with primary antibodies against STMN1 (1:200; Proteintech, 11157-1-AP), RAP1A (1:100; Abcam, ab197673), FLT3 (1:180; Abcam, ab52648), ANGPT2 (1:100; Proteintech, 24613-1-AP), PGF (1:200; Proteintech, 10642-1-AP), and HSPA8 (1:400; Proteintech, 10654-1-AP), CD276 (B7H3) (1:100; Abcam, ab105922), CD274 (PD-L1) (1:100; CST, #13684) at 4°C overnight. A secondary goat anti-rabbit antibody (1:200; ab205718, Abcam, UK) further incubated the slides at 37°C for 30 min. Binding of the primary antibodies was visualized *via* diaminobenzidine method (DAB, Boster Biological Technology, USA). Finally, these sections were counterstained with hematoxylin, dehydrated by a gradient ethanol, followed by xylene, and mounted. The staining of each specimen was evaluated through two independent investigators blinded to the clinicopathological information.

When it was present in the membrane or cytoplasm, the expression of proteins was considered positive. The staining score was assessed according to parameters of intensity and extension, and scored to determine the protein expression profiles. The staining-based expression levels were divided into four categories: positive, moderate, low, and negative; the scoring system used is based on the proportion of stained cells (>75, 25–75, or <25%); and the intensity of staining was also categorized into four: strong, moderate, weak, or negative. A final combined score of 0–12 was obtained by multiplying the intensity and percentage scores. Patients were classified into high or low protein expression groups based on median expression scores.

### Real-Time Quantitative Polymerase Chain Reaction

Tumor specimens and adjacent normal tissues were collected from recruited HCC patients. Total RNAs were extracted from tissues with TRIzol reagent (Invitrogen, Carlsbad, CA, USA). About 500 ng of purified RNA was reverse transcribed into complementary DNAs (cDNAs) using SuperScript ™II (Life Technologies, Darmstadt, Germany), and underwent SYBR green-based real-time polymerase chain reaction using a standard protocol with specific primers (Applied Biosystems, Carlsbad, CA, USA). In the PCR amplification process, samples were predenaturated at 95°C for 10 min, then 40 cycles of denaturation at 95°C, and annealing at 60°C for 1 min. The 2^−ΔΔCT^ method was used to calculate the results, which are presented as the x-fold increase with the adjacent normal tissues as control. All primer sequences used in our study are the following:

ERK forward, 5′-TGGATTCCCTGGTTCTCTCTAAAG-3′; reverse, 5′-GGGTCTGTTTTCCGAGGATGA-3′; JNK forward, 5′-TGTGTGGAATCAAGCACCTTC-3′; reverse, 5′-AGGCGTCATCATAAAACTCGTTC-3′; RAP1A forward, 5′-TGTCTCACTGCACCTTCA-3′; reverse, 5′-GACTTCCCAACG CCTCCT-3′; P38 forward, 5′-CGACTTGCTGGAGAAGATGC-3′; reverse, 5′-GGCACAAAGCTGATGACTTC-3′.

### Western Blot

The total protein was extracted from four cell lines Huh7, HepG2, Hepa1-6 in ice by RIPA lysis buffer (Beyotime, Shanghai, China) with protease and phosphatase inhibitor cocktail (Roche, Basel, Switzerland). The protein concentration was detected by BCA Protein Assay Kit (Beyotime, Shanghai, China); proteins were separated by SDS-PAGE and transferred onto polyvinylidene difluoride membranes (Millipore, Bedford, MA). Then incubated in 5% Bovine Serum Albumin at room temperature for 2 h. The membranes were exposed to primary antibodies against ERK, p-ERK, JNK, p-JNK, p38, p-p38, and RAP1A (1:1,000 dilution; Santa Cruz Biotechnology, Santa Cruz, CA) or *β*-actin (1:1,000 dilution; Cell Signaling Technology, USA) at 4°C overnight. After that, the membranes were incubated with secondary antibody (1:5,000 dilution; Cell Signaling Technology, USA), and protein blots were detected by Automatic Chemiluminescence Imaging Analysis System (Tanon, Shanghai, China).

### Statistical Analyses

All statistical tests including Cox regression models, LASSO regression, ROC curve analysis and K–M survival analyses were conducted using Rversion 3.5.1 (https://www.r-project.org/). Statistical differences between distributions were computed by independent t test between two groups and Kruskal–Wallis for multigroup comparison. P <0.05 was considered statistically significant. The charts, forest plots, and calibration plots were drawn using R language. This study finally selected to build the diagnostic prediction model or diagnosis guidelines using a logistic regression method.

## Results

### Construction of A Nomogram for Predicting Prognostic Risk of MAPK-RAP1A

**Figure 1:**
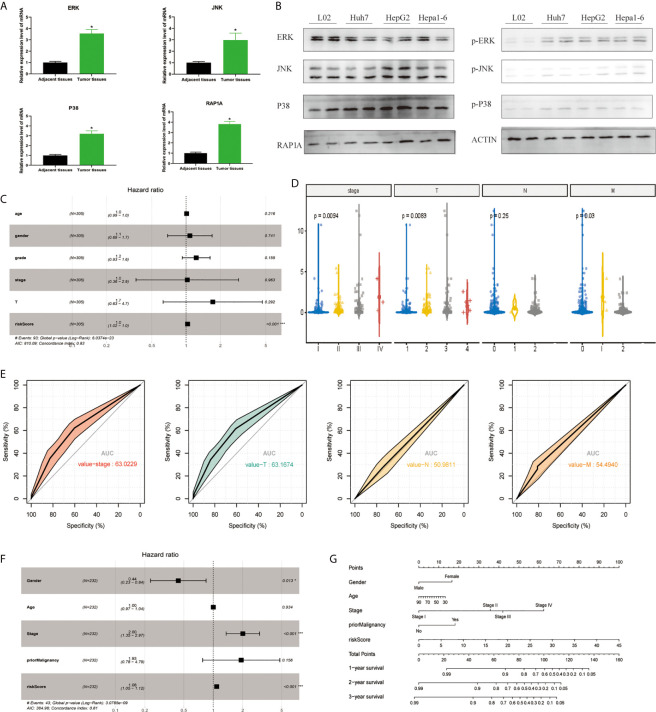
Establishment of the risk signature with MAPK-RAP1A related genes in the training (TCGA) and validation database (ICGC). **(A)** RT-qPCR was used to detect the expression of ERK, JNK, p38, RAP1A in 20 cases HCC tumor tissues and adjacent tissues. **(B)** Western blot was used to detect the expression of ERK, JNK, p38, RAP1A in Huh7, HepG2, Hepa1-6, and L02 cell lines. **(C)** Multivariate Cox regression analysis to validate prognosis related clinicopathological characters. **(D)** The correlation of our MAPK-RAP1A risk signature with the clinicopathological characters (TNM stage) of HCC from the training set. **(E)** ROC curves of AUC evaluated the efficiency of the risk signature for predicting TNM stage in training set. **(F)** Multivariate Cox regression analysis to validate prognosis related clinicopathological characters. **(G)** Nomogram for predicting 1-, 3-, and 5-year OS of LIHC patients in the ICGC cohort. *p < 0.05; ***p < 0.001.

In order to detect the MAPK-RAP1A pathway, we detected the expression of ERK, JNK, p38, and RAP1A by tissue RT-qPCR and found that the expression of ERK, JNK, p38, and RAP1A in HCC tumor tissues was higher than that in adjacent tissues (**Figure 1A**). We also detected the expression of ERK, JNK, p38, and RAP1A by western blot and found that the expression of phosphorylated ERK, JNK, p38, and RAP1A in Huh7, HepG2, and Hepa1-6 was higher than in L02 (**Figure 1B**). To better confirm the MAPK-RAP1A related genes with risk signature, we evaluated HCC patients with detailed clinical sample information (gender, age, histological grade, pathologic stage, and TNM stage) and follow-up information in the TCGA platform. Firstly, multivariate cox regression analyses were conducted to assess the independent predictive power in the training cohort, revealing that the risk signature remained as an independent risk factor correlated with HCC patients’ prognosis (*p* < 0.05) (**Figure 1C**). Second, risk signature was significantly correlated with TNM stages and overall survival (**Figures 1D, E** **)**. Subsequently, multivariate Cox regression analyses was tested in the validation cohort by the ICGC datasets, indicating that gender, stage, and risk scores were independent risk factors for HCC patients (*p* < 0.05) (**Figure 1F**). Finally, a nomogram integrating the factors was constructed for predicting clinical features of HCC. It was noted that the nomogram model demonstrated the probability accuracy for predicting HCC (**Figure 1G**). We considered that a nomogram integrating MAPK-RAP1A risk signature might act as accurate predictive power.

### Six-Related Genes Were Screened Out for Constructing a Risk Signature

**Figure 2:**
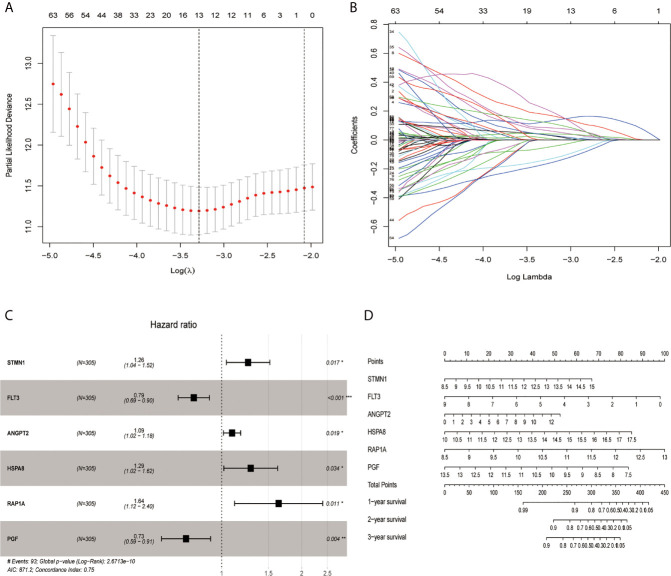
Screening out genes for constructing a risk signature. **(A)** Log (Lambda) value of the 11 genes in LASSO model. **(B)** The most proper log (Lambda) value in LASSO model. **(C)** Six genes (STMN1, FLT3, ANGPT2, HSPA8, RAP1A, and PGF) were selected for constructing a risk signature using multivariate Cox regression model. **(D)** A nomogram for predicting 1-, 3- and 5-year survival rate of HCC patients was established. *p < 0.05; **p < 0.01; ***p < 0.001.

Through the LASSO algorithm, 328 MAPK-RAP1A related gene were performed to build the prognostic risk signature in the TCGA training cohort, and 11 genes (STMN1, RAP1A, FLT3, HSPA8, FGF9, EFNA5, IRAK1, RAC3, DUSP10, ANGPT2, and PGF) were filtered out because of shrinking parameters (**Figure 2A**). Subsequently, six genes (STMN1, RAP1A, FLT3, HSPA8, ANGPT2, and PGF) were selected to establish a prognostic model (**Figure 2B**). Risk score = 1.5962 * (the expression level of STMN1) + 1.7459 * (the expression level of RAP1A) + 2.2693* (the expression level of FLT3) + 1.3980 * (the expression level of HSPA8) + 1.462 * (the expression level of ANGPT2) + 1.7673 * (the expression level of PGF). Finally, a nomogram integrating the six signatures was demonstrating the performance of the risk signature in predicting HCC (**Figure 2C**). The mentioned nomogram revealed ta better predictive accuracy by the total points obtained by adding up (**Figure 2D**).

### Stratification of TCGA Training and ICGC Validation Cohorts Using the Risk Signature

**Figure 3:**
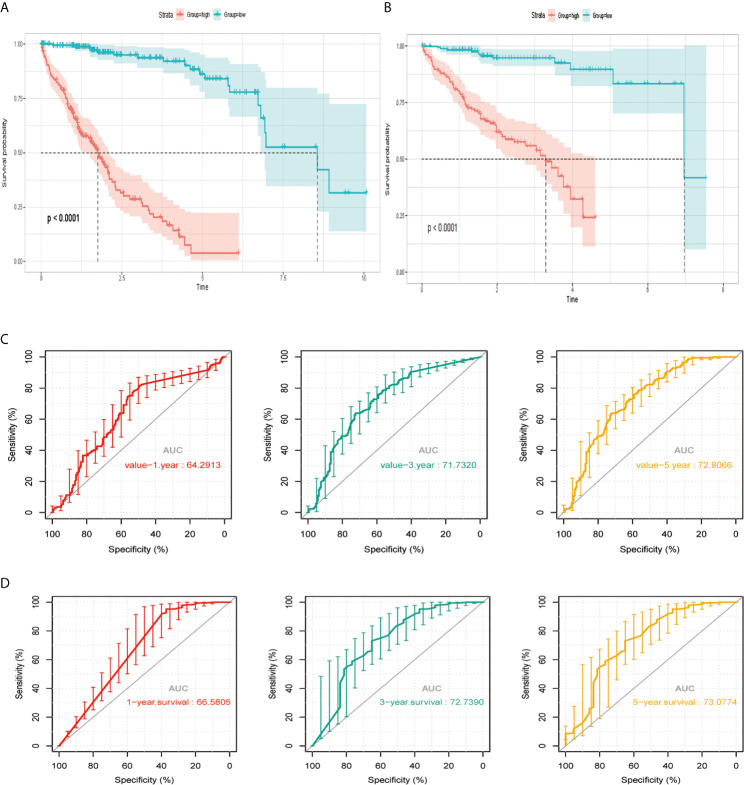
The risk signature in HCC from the training set, the independent external validation cohort. **(A, C)** The Kaplan–Meier curves of the MAPK-RAP1A related risk signature. **(B, D)** Time-dependent ROC curves at 1, 3, and 5 years. AUC, area under the ROC; HCC, hepatocellular carcinoma; ROC, receiver-operating characteristic.

According to the median risk score, we divided the cohort patients into high-risk and low-risk groups. The high-risk group exhibited a higher frequency of poorer overall survival (OS) than the low-risk group in the TCGA platform by K–M curve (**Figure 3A**). To further assess the predictive accuracy of this MAPK-RAP1A risk signature, we performed a time-dependent ROC curve analysis. The AUC was 0.75, 0.805, and 0.65 at 1, 3, and 5 years for survival in the training cohort of TCGA (**Figure 3C**). The ICGC dataset protocols were used for the validation of the risk signature for this purpose, which indicated that higher scores exhibited markedly worse overall survival (OS) (**Figure 3B**). The AUCs for OS in the validation cohort were 0.75, 0.805, and 0.65 at 1, 3, and 5 years, respectively (**Figure 3D**). Thus, we considered that the clinical prognostic model successfully stratified cohort patients from TCGA into high and low-risk groups with predicting the clinical prognosis of HCC.

### Validation of Signature Genes in the MAPK-RAP1A Datasets

**Figure 4:**
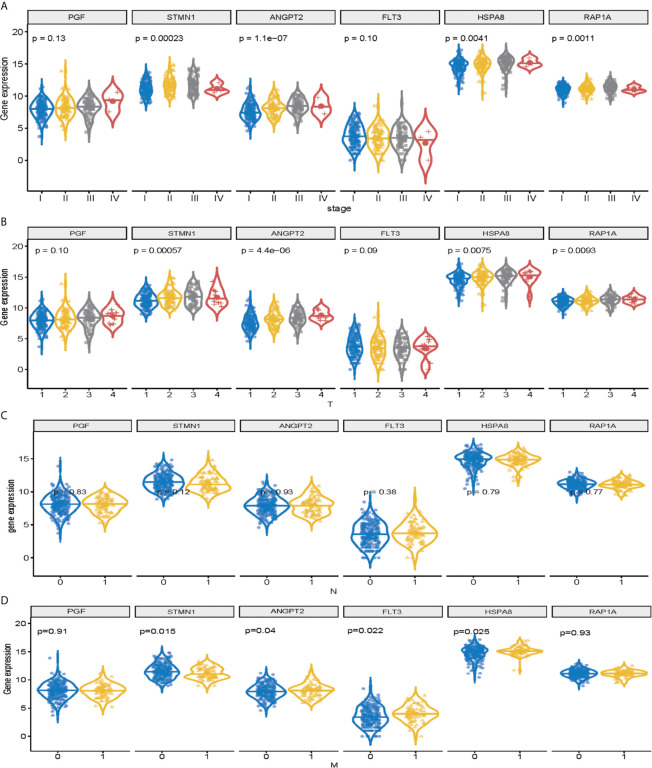
Validation of signature genes in the MAPK-RAP1A dataset. **(A)** Expression of STMN1, FLT3, ANGPT2, HSPA8, RAP1A, and PGF in HCC samples with different TNM stages. **(B)** Expression of STMN1, FLT3, ANGPT2, HSPA8, RAP1A, and PGF in HCC samples with different T stages. **(C)** Expression of STMN1, FLT3, ANGPT2, HSPA8, RAP1A, and PGF in HCC samples with different N stages. **(D)** Expression of STMN1, FLT3, ANGPT2, HSPA8, RAP1A, and PGF in HCC samples with different M stages.

Among the 11 genes screened above, six signature genes (STMN1, RAP1A, FLT3, HSPA8, ANGPT2, and PGF) were identified to validate prognostic and diagnostic value and clinical features. All of them, which were significantly upregulated (*p* < 0.05) in HCC samples, can potentially act as oncogenes compared with normal controls. In addition, STMN1, RAP1A, FLT3, HSPA8, ANGPT2, and PGF were identified as the key oncogenic components in HCC samples with different TNM stage, T classification, lymph node metastasis, and distant metastasis, with higher expression levels indicating higher stage (**Figures 4A–D**). Conversely, all six signature genes in the module might act as potential prognostic values.

### The Categories of Immunity and Tumor Purity of MAPK-RAP1A Datasets

**Figure 5:**
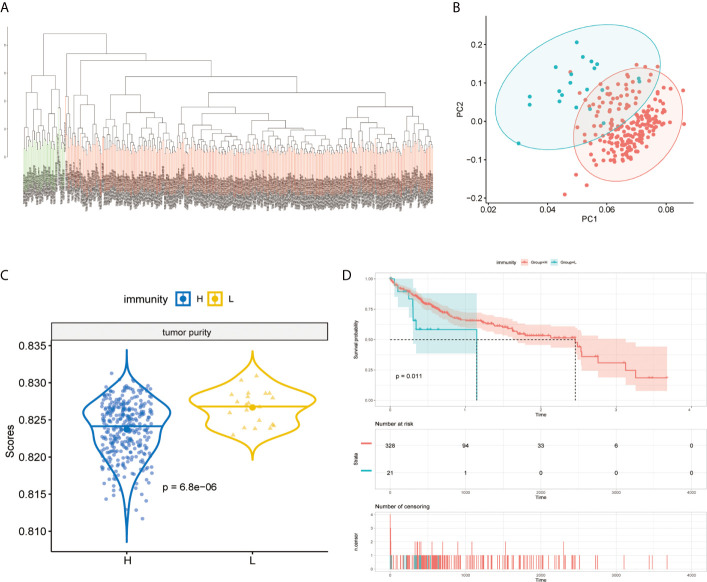
Establishment of the MAPK-RAP1A with immunity in the TCGA database. **(A, B)** PCA based on MAPK-RAP1A gene signature between low-immunity group and high-immunity group. **(C)** Tumor purity in different immunity subgroups. **(D)** Kaplan–Meier curves of overall survival according to immunity groups in the training cohort.

The immunity of the MAPK-RAP1A signaling was used in the principal components analysis (PCA) for assigning patients to low- and high-immunity categories (**Figures 5A, B** **)**. Further comparison analysis showed that tumor purity had significantly higher expression levels in low-immunity compared with high-immunity (**Figure 5C**). Regarding prognosis, K–M curves showed that higher tumor purity of MAPK-RAP1A signaling was associated significantly with worse OS (**Figure 5D**).

### Association of Stromal Score, Immune Score, and ESTIMATE Score With Several Clinical Features

**Figure 6:**
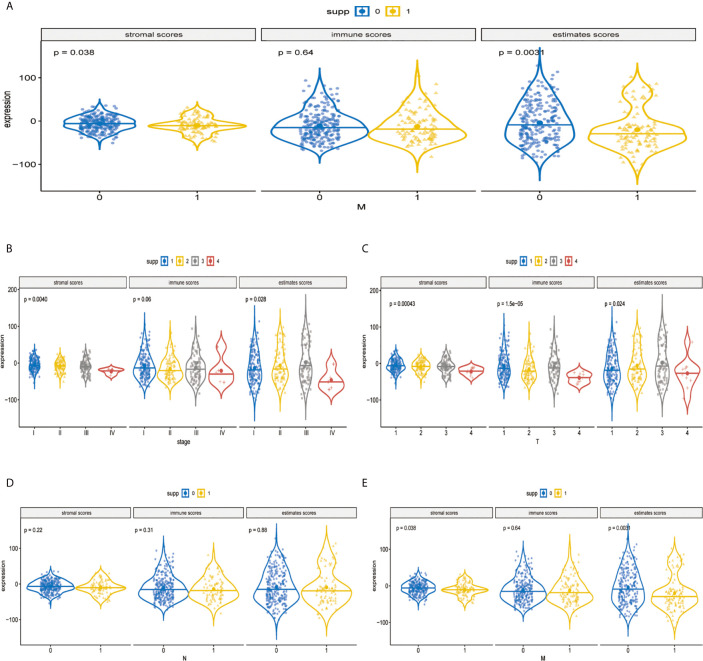
Validation of immune estimate in the MAPK-RAP1A dataset. **(A)** The corresponding stromal score, immune score, and estimated score of MAPK-RAP1A gene signature between low-immunity group and high-immunity group. **(B)** Scores of stromal, immune, and ESTIMATE in HCC samples with different TNM stages. **(C)** Scores of stromal, immune, and ESTIMATE in HCC samples with different T stages. **(D)** Scores of stromal, immune, and ESTIMATE in HCC samples with different N stages. **(E)** Scores of stromal, immune, and ESTIMATE in HCC samples with different M stages.

The immune components and clinicopathological characteristics were positively correlated; we analyzed the corresponding stromal score, immune score, and estimate score of MAPK-RAP1A gene sets from TCGA-LIHC database (**Figure 6A**). Among them, stromal score and estimate score are key proportion of advanced TNM stages. The representative images of the immune score were only negatively correlated with T classification of TMN stages (*p* = 0.007), and estimate score significantly declined with T and M classification of TMN stages (*p* = 0.028 and *p* = 0.021). Whereas there was no correlation between ESTIMATE algorithm and lymph node metastasis (*P* = 0.776). These results suggested that the ratio of immune components was correlated with the progress of HCC, especially in metastasis (**Figures 6B–E**).

### Association of Risk Signature With Tumor Purity and Tumor Immune Cell Infiltration

**Figure 7:**
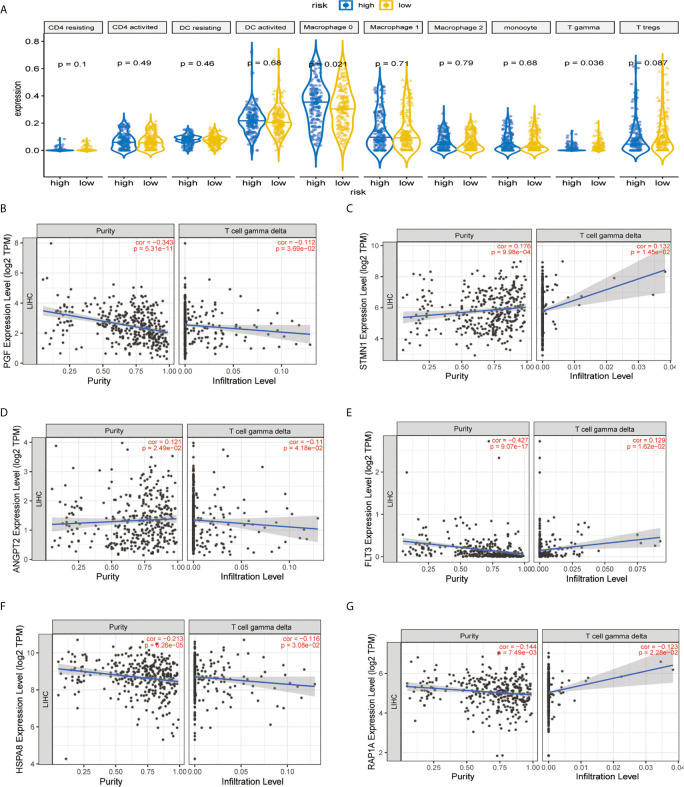
Immune infiltration construction and validation in HCC. **(A)** Plot for comparison of the immune cell fraction difference between high risk and low risk. Fractions of each immune cell type were compared. Association of hub genes’ expression with immune infiltration in HCC. **(B)** PGF **(C)** STMN1, **(D)** ANGPT2, **(E)** FLT3, **(F)** HSPA8, and **(G)** RAP1A. *P <*0.05 denotes significance. Each dot represents a sample in the TCGA-LIHC dataset.

It was demonstrated that the levels of TICs, including Tregs, T-cells gamma delta (V*δ* T cells), and monocytes were significantly higher in the normal patient group compared with the HCC patient group (**Figure 7A**). As shown in **Figure 7B**, two immune infiltration cell subpopulations were significantly enriched in the low-risk patient group (Macrophage 0 and V*δ* T cells). To characterize the TICs, the contents of infiltrating immune cells were investigated. We utilized TIMER website source to identified potential associations between the expression of signature genes and immune purity of tumor, infiltrating immune cells (**Figures 7C** **–G**). Interestingly, STMN1 and ANGPT2 were significantly positively correlated with tumor purity, whereas RAP1A, FLT3, HSPA8, and PGF were all significantly negatively correlated with tumor purity. Consequently, the present study reveals relationships linking these six genes and infiltration of V*δ* T cells (**Figures 7C–G**).

### GSEA Reveal a Close Relationship Between Hub Genes and Tumor Proliferation

**Figure 8:**
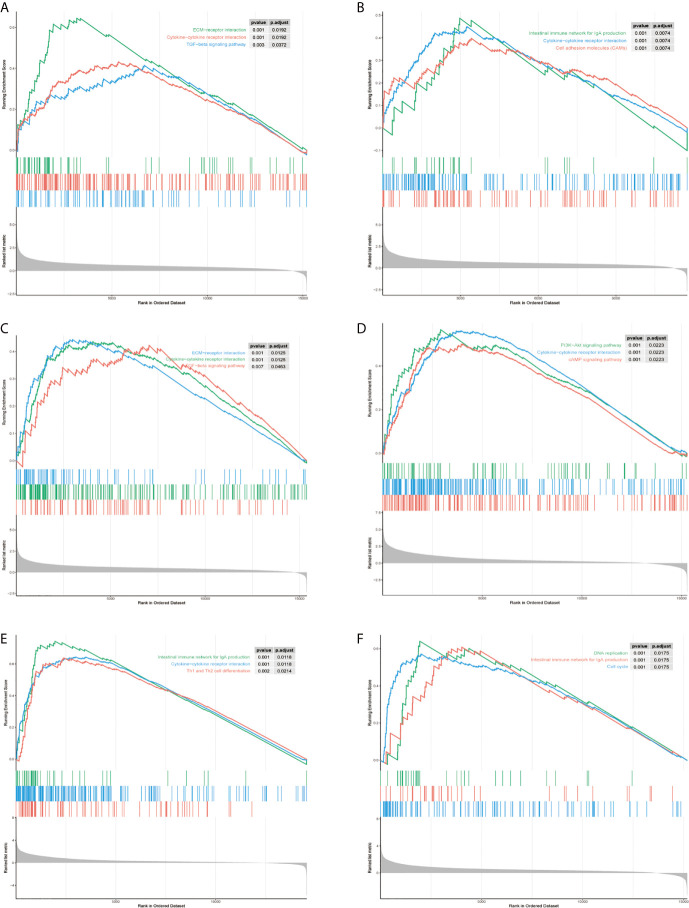
Gene set enrichment analysis (GSEA) of hub genes in the TCGA-LIHC MAPK-RAP1A dataset. **(A–F)** Top three gene sets (according to GSEA enrichment score) enriched in the high-expression group of single hub genes. **(A)** PGF, **(B)** STMN1, **(C)** ANGPT2, **(D)** FLT3, **(E)** HSPA8, and **(F)** RAP1A.

To explore the molecular mechanisms of STMN1, RAP1A, FLT3, HSPA8, ANGPT2, and PGF in HCC, we detected GSEA on the TCGA-LIHC RNA-seq data. This work confirmed that the role of hub genes in high expression groups of RAP1A, FLT3, HSPA8, ANGPT2, and PGF were all enriched in “cytokine−cytokine receptor interaction” pathways. Additionally, the “ECM−receptor interaction” and “TGF−beta signaling pathway” gene set were enriched in high expression groups of ANGPT2 and HSPA8, whereas “Intestinal immune network for IgA production” was enriched in the PGF, FLT3, and STMNI high-expression groups, respectively, which suggest that these hub gene sets were all closely involved in tumor proliferation (**Figures 8A–F**).

### Correlation of the Signature With B7 Therapy-Related Molecule

**Figure 9:**
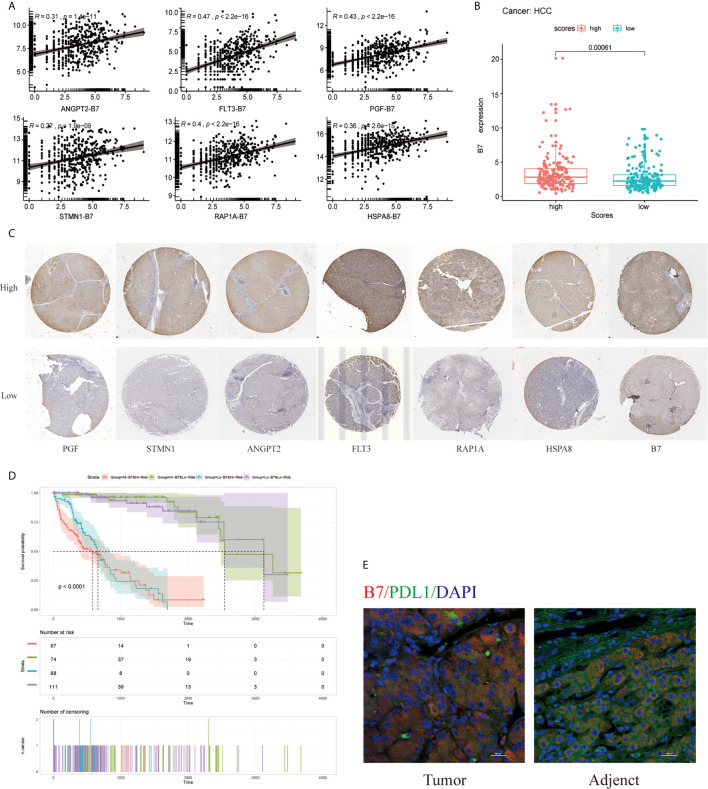
Effect of the risk signature and immune checkpoint gene expression on patient survival. **(A)** Association between the risk signature and immune checkpoint gene expression. **(B)** Association between the hub gene and immune checkpoint gene expression. **(C)** Immunohistochemical staining was used to compare the expression of PGF, STMN1, ANGPT2, FLT3, RAP1A, HSPA8 with B7 in HCC tissues. **(D)** Kaplan–Meier survival curves of OS among patient groups stratified by the risk signature and immune checkpoint gene expression. **(E)** Association between B7 (CD80) and PDL1 in HCC tissues compared with adjacent tissues. Supplemental Figure Immunohistochemistry was used to detect the negative control, high and low expression of PGF, STMN1, ANGPT2, FLT3, RAP1A, HSPA8, and B7 in HCC tissues at high and low magnification.

Moreover, B7(CD80) served as specific markers for T cell gamma delta (V*δ* T), respectively. As shown in **Figure 9A**, the hub genes of ANGPT2, FLT3, PGF, STMN1, RAP1A, and HSPA8 were significantly positively correlated with immune checkpoint B7(CD80) in the TCGA database (*p* =1.41e-11 for ANGPT2, *p <*2.2e-16 for FLT3, *p <*2.2e-16 for PGF, *p <*1.9e-9 for STMN1, *p <*2.2e-16 for RAP1A, *p* =2.6e-15 for HSPA8) (**Figure 9A**). Subsequently, the relationships between the risk signature with MAPK-RAP1A signaling and the expression levels of B7 were found. The data here indicated that higher immune checkpoint gene of B7 may be significantly observed in high-risk patients (*p* = 0.012) (**Figure 9B**). Interestingly, the expression levels of PGF, STMN1, ANGPT2, FLT3, RAP1A, and HSPA8 were all significant positively correlated with B7 in HCC tissues (**Figure 9C**) (**Supplement Figure**). To further access the prognostic model between risk signature and B7 on patients’ OS, a survival comparison was made among the four groups based on the combination of risk signature and immune checkpoint gene of B7. Comparison results revealed that HCC patients with risk signature and B7 in the predictive models are able to distinguish the overall survival (*p <*0.0001) (**Figure 9D**). Finally, the present study reveals relationships linking B7(CD80) and PDL1 in HCC tissues compared with adjacent tissues (**Figure 9E**).

## Discussion

In the current study, a novel network was developed that enabled the identification of the MAPK-RAP1A risk signature for the evaluation of clinical feature, and its clinical feature was associated with tumor-infiltrating immune cells (TICs). As expected, KEGG pathways were mostly concentrated on MAPK and RAP1A signaling. The reliable and effective MAPK-RAP1A risk signature accurately predicts the diagnosis of HCC patients. Computation analysis among TICs of MAPK-RAP1A risk signature, were preferentially observed in T cell gamma delta compared with other immune cells. Based on the analysis of TCGA and ICGC dataset, a risk signature was identified. The risk signature was composed of six signature genes which were screened out from 374 cases of HCC and 50 normal tissues. To characterize the results of the GSEA, the signature genes were associated with immune and inflammation mechanisms.

In order to further elucidate the role of the risk signatures in cancer, the association of the risk signature and clinical model was assessed. For example, a recent study indicated that the immune related nine-gene signature is involved in the status of tumor immune microenvironment (26). Another study also constructed a known lncRNAs prognostic signature for cancer and may aid in the field of tumor immunity and immunotherapy (27). Studies conducted so far concluded that investigating the prognostic value of tumor-infiltrating B lymphocytes lncRNA signature (TILBlncSig) in bladder cancer identified and validated biomarkers of immunotherapy response (28). However, these studies did not use the signaling pathway of samples to comprehensively explore the relationship between TICs and prognosis of HCC.

To probe the signaling events in HCC expression, this is the first study to reveal the relationships linking MAPK-RAP1A signaling pathway and TICs. We obtained a number of prognostic MAPK-RAP1A signature genes and established a novel patient prognosis and clinical feature model. Consistently, we have linked prognostic model to poor OS, which is consistent with other studies in HCC. Consequently, the MAPK-RAP1A related prognostic model provides a more reliable tool, which was a good prognostic factor with HCC. In addition, such model that consists of six signature genes was then successfully validated as a prognostic factor. Based on this overall hypothesis, we revealed that MAPK-RAP1A prognostic model could act as a more reliable tool for HCC prognosis prediction.

In addition, we identified MAPK-RAP1A signature genes (STMN1, RAP1A, FLT3, HSPA8, ANGPT2 and PGF) which were related with HCC worse clinical phenotype and prognosis. STMN1 is an oncogene that is a highly conserved cytosolic phosphoprotein, was found to be over-expressed in various types of cancer such as lung, breast, gastric cancer, and HCC. It plays an important role in cell differentiation, proliferation, drug resistance, and cancer stem cell properties and as an emerging target for tumor therapy (29). STMN1 regulated cancer-associated fibroblast (CAF) features through HSC by triggering the HGF/MET signaling pathway (30).

RAP1A is a small G protein that is similar to Ras oncogene and associated with different cellular processes. Other studies also showed that RAP1A may be a potential target for cell proliferation, adhesion, and invasion in different types of cancers (9, 31, 32). Recent clinical studies reported that RAP1A also correlated with the clinical characteristics of the advanced tumor stage in Oral Cavity Squamous Cell Carcinoma (OCSCC) (33). Another study showed that RAP1A disrupts aberrant tumor suppressor of EYA4 in HCC cells, which was associated with promoting growth and invasion (34).

FLT-3 belongs to the receptor tyrosine kinase family, which is encoded by the FLT3 gene. FLT3 has been found to control the function of normal and malignant hematopoiesis (34). Activating mutations of FLT3 associated with a poor prognosis in acute myeloid leukemia (AML) and FLT3 inhibitors have an important role in high-risk patients (35). Targeting a tyrosine kinase receptor, sorafenib is now routinely required to provide therapy benefits in FLT3 of HCC patients (36, 37). Moreover, the association between patients treated with sorafenib and high FLT3 levels could be a novel predictor to improve OS in HCC patients (38).

The HSPA8 protein integrates compensatory mechanisms to drive cellular growth and is dysregulated in multiple chronic stress diseases, including cancer. Mechanistically, the molecular chaperone HSPA8, also known as Hsc70c, and directed to lysosomes, selectively degrades cellular proteins to sustain cellular homeostasis (39). The role of HSPA8 is expressed abnormally in early liver cancer, and its expression increases with cancer initiation and progression (40). So, HSPA8 detection may improve early detection of liver cancer sensitive indicators, but whether and how HSPA8 is involved in cancer initiation and development has been explored.

ANGPT2 is available in the extracellular signals that play a crucial role in angiogenesis and resistance to antiangiogenic therapy (41, 42). ANGPT2 is an angiogenic factor that binds Tie2 receptor and emerged as an attractive vascular drug target with the vascular endothelial growth factor (VEGF) pathway (43). Currently, ANGPT2 has been playing an important role in tumor angiogenesis and might confer resistance to sorafenib therapy (44–46). Furthermore, a study reveals that HCC cell secreted exosomal ANGPT2 was recycled by recipient HUVECs that suppressed the epithelial–mesenchymal transition (EMT) activation (47).

Placenta growth factor (PGF), a member of the VEGF family, also plays an important role in the current anti-angiogenesis therapy. In this way, the present study has generated that patients with higher PGF had poorer response to chemotherapy and poorer prognosis and identified biomarkers in epithelial ovarian cancer (EOC) (48). PGF has been involved in elevated NF-kB signaling pathway in cervical cancer (49), but its role in HCC remains unclear.

To characterize the tumor-infiltrating immune cells’ (TICs’) status, the relationships between risk signature model and immune cell were analyzed by GSEA and CIBERSORT. The high abundance of TICs for risk signature may also affect clinical features and survival analysis. In addition, the data here indicated that higher infiltration levels of T cell gamma delta (V*δ* T cells) were independent prognostic protective factors in HCC. Similar to previous reports (50, 51), our data confirmed that the infiltration of V*δ* T cells was an independent prognostic factor. V*δ* T cells play a critical role in the solid tumor microenvironment, but the effectiveness in killing various tumor cells present has been shown to be limited (50–52). V*δ*2 T cells infiltrate several types of tumors, including the liver, and could serve as a prognostic factor (53). Activated V*δ*2 T cells can exhibit the functions and characteristics of dendritic cells (54). On the one hand, V*δ*2 T cells not only express the chemokine receptor CCR7 on the surface, but also upregulate the expression levels of MHCI and MHCII molecules, as well as co-stimulating molecules CD80 (B7) and CD86 (55).

In order to elucidate the role of the signature genes’ biological functions, the association of STMN1, RAP1A, FLT3, HSPA8, ANGPT2, and PGF was positively associated with tumor purity in HCC. Based on the exploration from TIMER website, of the risk signature, six signature genes (STMN1, RAP1A, FLT3, HSPA8, ANGPT2, and PGF) were associated with infiltrating immune cells. Characterization of immune regulation by the GSEA indicated significantly different immune function among different expression groups classified by the hub gene. The results of GSEA were in accordance with this speculation. Also, many immune-related KEGG pathways, such as cytokine−cytokine receptor interaction, ECM−receptor interaction and TGF−beta signaling pathway, suggested a signature contribution to immunity regulation.

In summary, taken together, all the above research suggested that the MAPK-RAP1A risk signature maybe a potential prognostic molecular marker for HCC patients. Furthermore, the predictive value of the risk signature was validated in available clinical information and RNAseq data. The MAPK-RAP1A risk signature was not only shown to have prognostic value for HCC patients but was also related to immune cell infiltration (T cell gamma delta) and the immunotherapy signature. The MAPK-RAP1A risk signature added significant predictive power to CD80 (B7) expression, which led to a significant overall survival. Altogether, these results suggested that the risk signature could shed light on the mechanisms of immunotherapeutic targeting.

## Data Availability Statement

The datasets presented in this study can be found in online repositories. The names of the repository/repositories and accession number(s) can be found in the article/**Supplementary Material**.

## Ethics Statement

The studies involving human participants were reviewed and approved by the research ethics committee of Hongqi Hospital Affiliated to Mudanjiang Medical University. The patients/participants provided their written informed consent to participate in this study. Written informed consent was obtained from the individual(s) for the publication of any potentially identifiable images or data included in this article.

## Author Contributions

HL, GH, XL, BL, and LW contributed to the bioinformatics analysis. BW and HJ performed the experiment. WW reviewed the article and provided comments. All authors contributed to the article and approved the submitted version.

## Conflict of Interest

The authors declare that the research was conducted in the absence of any commercial or financial relationships that could be construed as a potential conflict of interest.

## References

[1] Llovet JM Zucman-Rossi J Pikarsky E Sangro B Schwartz M Sherman M. Hepatocellular Carcinoma. Nat Rev Dis Primers (2016) 2:16018. 10.1038/nrdp.2016.18 27158749

[2] Allemani C Weir HK Carreira H Harewood R Spika D Wang XS. Global Surveillance of Cancer Survival 1995-2009: Analysis of Individual Data for 25,676,887 Patients From 279 Population-Based Registries in 67 Countries (CONCORD-2). Lancet (2015) 385(9972):977–1010. 10.1016/S0140-6736(14)62038-9 25467588 PMC4588097

[3] Joliat GR Allemann P Labgaa I Demartines N Halkic N. Treatment and Outcomes of Recurrent Hepatocellular Carcinomas. Langenbecks Arch Surg (2017) 402(5):737–44. 10.1007/s00423-017-1582-9 28497194

[4] Bruix J Gores GJ Mazzaferro V. Hepatocellular Carcinoma: Clinical Frontiers and Perspectives. Gut (2014) 63(5):844–55. 10.1136/gutjnl-2013-306627 PMC4337888 24531850

[5] Lin DC Mayakonda A Dinh HQ Huang P Lin L Liu X. Genomic and Epigenomic Heterogeneity of Hepatocellular Carcinoma. Cancer Res (2017) 77(9):2255–65. 10.1158/0008-5472.CAN-16-2822 PMC5413372 28302680

[6] Wagner ER Nebreda AR. Signal Integration by JNK and P38 MAPK Pathways in Cancer Development. Nat Rev Cancer (2009) 9(8):537–49. 10.1038/nrc2694 19629069

[7] Nussinov R Jang H Zhang M Tsai CJ Sablina AA. The Mystery of Rap1 Suppression of Oncogenic Ras. Trends Cancer (2020) 6(5):369–79. 10.1016/j.trecan.2020.02.002 PMC7211489 32249186

[8] Cowan KJ Storey KB. Mitogen-Activated Protein Kinases: New Signaling Pathways Functioning in Cellular Responses to Environmental Stress. J Exp Biol (2003) 206(Pt 7):1107–15. 10.1242/jeb.00220 12604570

[9] Xiang J Bian C Wang H Huang S Wu D. MiR-203 Down-Regulates Rap1A and Suppresses Cell Proliferation, Adhesion and Invasion in Prostate Cancer. J Exp Clin Cancer Res (2015) 34:8. 10.1186/s13046-015-0125-x 25636908 PMC4321708

[10] Anuja K Kar M Chowdhury AR Shankar G Padhi S Roy S. Role of Telomeric RAP1 in Radiation Sensitivity Modulation and its Interaction With CSC Marker KLF4 in Colorectal Cancer. Int J Radiat Biol (2020) 96(6):790–802. 10.1080/09553002.2020.1721609 31985344

[11] Gaonac’h-Lovejoy V Boscher C Delisle C Gratton JP. Rap1 is Involved in Angiopoietin-1-Induced Cell-Cell Junction Stabilization and Endothelial Cell Sprouting. Cells (2020) 9(1):15. 10.3390/cells9010155 PMC7016689 31936361

[12] Lu L Wang J Wu Y Wan P Yang G. Rap1A Promotes Ovarian Cancer Metastasis Via Activation of ERK/p38 and Notch Signaling. Cancer Med (2016) 5(12):3544–54. 10.1002/cam4.946 PMC5224839 27925454

[13] Angell H, Galon J. From the Immune Contexture to the Immunoscore: The Role of Prognostic and Predictive Immune Markers in Cancer. Curr Opin Immunol (2013) 25(2):261–7. 10.1016/j.coi.2013.03.004 23579076

[14] Gentles AJ Newman AM Liu CL Bratman SV Feng W Kim D. The Prognostic Landscape of Genes and Infiltrating Immune Cells Across Human Cancers. Nat Med (2015) 21(8):938–45. 10.1038/nm.3909 PMC4852857 26193342

[15] Grivennikov SI Greten FR Karin M. Immunity, Inflammation, and Cancer. Cell (2010) 140(6):883–99. 10.1016/j.cell.2010.01.025 PMC2866629 20303878

[16] Shin MH Kim J Lim SA Kim J Lee KM. Current Insights Into Combination Therapies With MAPK Inhibitors and Immune Checkpoint Blockade. Int J Mol Sci (2020) 21(7):2531. 10.3390/ijms21072531 PMC7177307 32260561

[17] Shen T Huang Z Shi C Pu X Xu X Wu Z. Pancreatic Cancer-Derived Exosomes Induce Apoptosis of T Lymphocytes Through the P38 MAPK-mediated Endoplasmic Reticulum Stress. FASEB J (2020) 34(6):8442–58. 10.1096/fj.201902186R 32350913

[18] Ye L Zhang Q Cheng Y Chen X Wang G Shi M. Tumor-Derived Exosomal HMGB1 Fosters Hepatocellular Carcinoma Immune Evasion by Promoting TIM-1(+) Regulatory B Cell Expansion. J Immunother Cancer (2018) 6(1):145. 10.1186/s40425-018-0451-6 30526680 PMC6288912

[19] Xiong Y Ye C Yang N Li M Liu H. Ubc9 Binds to ADAP and Is Required for Rap1 Membrane Recruitment, Rac1 Activation, and Integrin-Mediated T Cell Adhesion. J Immunol (2017) 199(12):4142–54. 10.4049/jimmunol.1700572 29127148

[20] Tibshirani R. The Lasso Method for Variable Selection in the Cox Model. Stat Med (1997) 16(4):385–95. 10.1002/(SICI)1097-0258(19970228)16:4<385::AID-SIM380>3.0.CO;2-3 9044528

[21] Subramanian A Tamayo P Mootha VK Mukherjee S Ebert BL Gillette MA. Gene Set Enrichment Analysis: A Knowledge-Based Approach for Interpreting Genome-Wide Expression Profiles. Proc Natl Acad Sci U S A (2005) 102(43):15545–50. 10.1073/pnas.0506580102 PMC1239896 16199517

[22] Newman AM Liu CL Green MR Gentles AJ Feng W Xu Y. Robust Enumeration of Cell Subsets From Tissue Expression Profiles. Nat Methods (2015) 12(5):453–7. 10.1038/nmeth.3337 PMC4739640 25822800

[23] Li B Severson E Pignon JC Zhao H Li T Novak J. Comprehensive Analyses of Tumor Immunity: Implications for Cancer Immunotherapy. Genome Biol (2016) 17(1):174. 10.1186/s13059-016-1028-7 27549193 PMC4993001

[24] Li T Fan J Wang B Traugh N Chen Q Liu JS. Timer: A Web Server for Comprehensive Analysis of Tumor-Infiltrating Immune Cells. Cancer Res (2017) 77(21):e108–10. 10.1158/0008-5472.CAN-17-0307 PMC6042652 29092952

[25] Yu G Wang LG Han Y He QY. clusterProfiler: An R Package for Comparing Biological Themes Among Gene Clusters. OMICS (2012) 16(5):284–7. 10.1089/omi.2011.0118 PMC3339379 22455463

[26] Wang Z Zhu J Liu Y Liu C Wang W Chen F. Development and Validation of a Novel Immune-Related Prognostic Model in Hepatocellular Carcinoma. J Transl Med (2020) 18(1):67. 10.1186/s12967-020-02255-6 32046766 PMC7011553

[27] Xu S Wang Q Kang Y Liu J Yin Y Liu L. Long Noncoding Rnas Control the Modulation of Immune Checkpoint Molecules in Cancer. Cancer Immunol Res (2020) 8(7):937–51. 10.1158/2326-6066.CIR-19-0696 32321773

[28] Zhou M Zhang Z Bao S Hou P Yan C Su J Sun J. Computational Recognition of lncRNA Signature of Tumor-Infiltrating B Lymphocytes With Potential Implications in Prognosis and Immunotherapy of Bladder Cancer. Brief Bioinform (2020) 2020:bbaa047. 10.1093/bib/bbaa047 32382761

[29] Hsieh SY Huang SF Yu MC Yeh TS Chen TC Lin YJ. Stathmin1 Overexpression Associated With Polyploidy, Tumor-Cell Invasion, Early Recurrence, and Poor Prognosis in Human Hepatoma. Mol Carcinog (2010) 49(5):476–87. 10.1002/mc.20627 20232364

[30] Zhang R Gao X Zuo J Hu B Yang J Zhao J. STMN1 Upregulation Mediates Hepatocellular Carcinoma and Hepatic Stellate Cell Crosstalk to Aggravate Cancer by Triggering the MET Pathway. Cancer Sci (2020) 111(2):406–17. 10.1111/cas.14262 PMC7004522 31785057

[31] Sayyah J Bartakova A Nogal N Quilliam LA Stupack DG Brown JH. The Ras-related Protein, Rap1A, Mediates Thrombin-Stimulated, Integrin-Dependent Glioblastoma Cell Proliferation and Tumor Growth. J Biol Chem (2014) 289(25):17689–98. 10.1074/jbc.M113.536227 PMC4067203 24790104

[32] Chen CH Chuang HC Huang CC Fang FM Huang HY Tsai HT. Overexpression of Rap-1A Indicates a Poor Prognosis for Oral Cavity Squamous Cell Carcinoma and Promotes Tumor Cell Invasion Via Aurora-A Modulation. Am J Pathol (2013) 182(2):516–28. 10.1016/j.ajpath.2012.10.023 23219753

[33] Mo SJ Hou X Hao XY Cai JP Liu X Chen W. EYA4 Inhibits Hepatocellular Carcinoma Growth and Invasion by Suppressing NF-kappaB-dependent RAP1 Transactivation. Cancer Commun (Lond) (2018) 38(1):9. 10.1186/s40880-018-0276-1 29764501 PMC5993152

[34] Stirewalt DL Radich JP. The Role of FLT3 in Haematopoietic Malignancies. Nat Rev Cancer (2003) 3(9):650–65. 10.1038/nrc1169 12951584

[35] Perl AE. Availability of FLT3 Inhibitors: How do We Use Them? Blood (2019) 134(9):741–5. 10.1182/blood.2019876821 31243041

[36] Levis M. Midostaurin Approved for FLT3-mutated Aml. Blood (2017) 129(26):3403–6. 10.1182/blood-2017-05-782292 28546144

[37] Daver N Schlenk RF Russell NH Levis MJ. Targeting FLT3 Mutations in AML: Review of Current Knowledge and Evidence. Leukemia (2019) 33(2):299–312. 10.1038/s41375-018-0357-9 30651634 PMC6365380

[38] Sun W Li SC Xu L Zhong W Wang ZG Pan CZ. High FLT3 Levels May Predict Sorafenib Benefit in Hepatocellular Carcinoma. Clin Cancer Res (2020) 26(16):4302–12. 10.1158/1078-0432.CCR-19-1858 32332018

[39] Robert G Jacquel A Auberger P. Chaperone-Mediated Autophagy and Its Emerging Role in Hematological Malignancies. Cells (2019) 8(10):1260. 10.3390/cells8101260 PMC6830112 31623164

[40] Xiang X You XM Li LQ. Expression of HSP90AA1/HSPA8 in Hepatocellular Carcinoma Patients With Depression. Onco Targets Ther (2018) 11:3013–23. 10.2147/OTT.S159432 PMC5973353 29872313

[41] Valenzuela DM Griffiths JA Rojas J Aldrich TH Jones PF Zhou H. Angiopoietins 3 and 4: Diverging Gene Counterparts in Mice and Humans. Proc Natl Acad Sci U S A (1999) 96(5):1904–9. 10.1073/pnas.96.5.1904 PMC26709 10051567

[42] Maisonpierre PC Suri C Jones PF Bartunkova S Wiegand SJ Radziejewski C. Angiopoietin-2, a Natural Antagonist for Tie2 That Disrupts In Vivo Angiogenesis. Science (1997) 277(5322):55–60. 10.1126/science.277.5322.55 9204896

[43] Jayson GC Kerbel R Ellis LM Harris AL. Antiangiogenic Therapy in Oncology: Current Status and Future Directions. Lancet (2016) 388(10043):518–29. 10.1016/S0140-6736(15)01088-0 26853587

[44] Morse MA Sun W Kim R He AR Abada PB Mynderse M. The Role of Angiogenesis in Hepatocellular Carcinoma. Clin Cancer Res (2019) 25(3):912–20. 10.1158/1078-0432.CCR-18-1254 30274981

[45] Marisi G Petracci E Raimondi F Faloppi L Foschi FG Lauletta G. ANGPT2 and NOS3 Polymorphisms and Clinical Outcome in Advanced Hepatocellular Carcinoma Patients Receiving Sorafenib. Cancers (Basel) (2019) 11(7):1023. 10.3390/cancers11071023 PMC6679015 31330833

[46] Scholz A Plate KH Reiss Y. Angiopoietin-2: A Multifaceted Cytokine That Functions in Both Angiogenesis and Inflammation. Ann N Y Acad Sci (2015) 1347:45–51. 10.1111/nyas.12726 25773744

[47] Xie JY Wei JX Lv LH Han QF Yang WB Li GL. Angiopoietin-2 Induces Angiogenesis Via Exosomes in Human Hepatocellular Carcinoma. Cell Commun Signal (2020) 18(1):46. 10.1186/s12964-020-00535-8 32183816 PMC7077328

[48] Meng Q Duan P Li L Miao Y. Expression of Placenta Growth Factor is Associated With Unfavorable Prognosis of Advanced-Stage Serous Ovarian Cancer. Tohoku J Exp Med (2018) 244(4):291–6. 10.1620/tjem.244.291 29643276

[49] Tilborghs S Corthouts J Verhoeven Y Arias D Rolfo C Trinh XB The Role of Nuclear Factor-Kappa B Signaling in Human Cervical Cancer. Crit Rev Oncol Hematol (2017) 120:141–50. 10.1016/j.critrevonc.2017.11.001 29198328

[50] Gebhardt T Mueller SN Heath WR Carbone FR. Peripheral Tissue Surveillance and Residency by Memory T Cells. Trends Immunol (2013) 34(1):27–32. 10.1016/j.it.2012.08.008 23036434

[51] Chien YH Meyer C Bonneville M. Gammadelta T Cells: First Line of Defense and Beyond. Annu Rev Immunol (2014) 32:121–55. 10.1146/annurev-immunol-032713-120216 24387714

[52] Fisher J Anderson J. Engineering Approaches in Human Gamma Delta T Cells for Cancer Immunotherapy. Front Immunol (2018) 9:1409. 10.3389/fimmu.2018.01409 29997614 PMC6028554

[53] Lo Presti E Pizzolato G Corsale AM Caccamo N Sireci G Dieli F. Gammadelta T Cells and Tumor Microenvironment: From Immunosurveillance to Tumor Evasion. Front Immunol (2018) 9:1395. 10.3389/fimmu.2018.01395 29963061 PMC6013569

[54] Willcox CR Davey MS Willcox BE. Development and Selection of the Human Vgamma9vdelta2(+) T-Cell Repertoire. Front Immunol (2018) 9:1501. 10.3389/fimmu.2018.01501 30013562 PMC6036166

[55] Dar AA Patil RS Patil RS and Chiplunkar SV. Insights Into the Relationship Between Toll Like Receptors and Gamma Delta T Cell Responses. Front Immunol (2014) 5:366. 10.3389/fimmu.2014.00366 25132835 PMC4116803

