# Supplementary figures and images for: MAPK-RAP1A Signaling Enriched in Hepatocellular Carcinoma Is Associated With Favorable Tumor-Infiltrating Immune Cells and Clinical Prognosis
Source: Front Oncol. 2021 Jun 10;11:649980. doi: 10.3389/fonc.2021.649980 (PMC8222816; doi:10.3389/fonc.2021.649980)

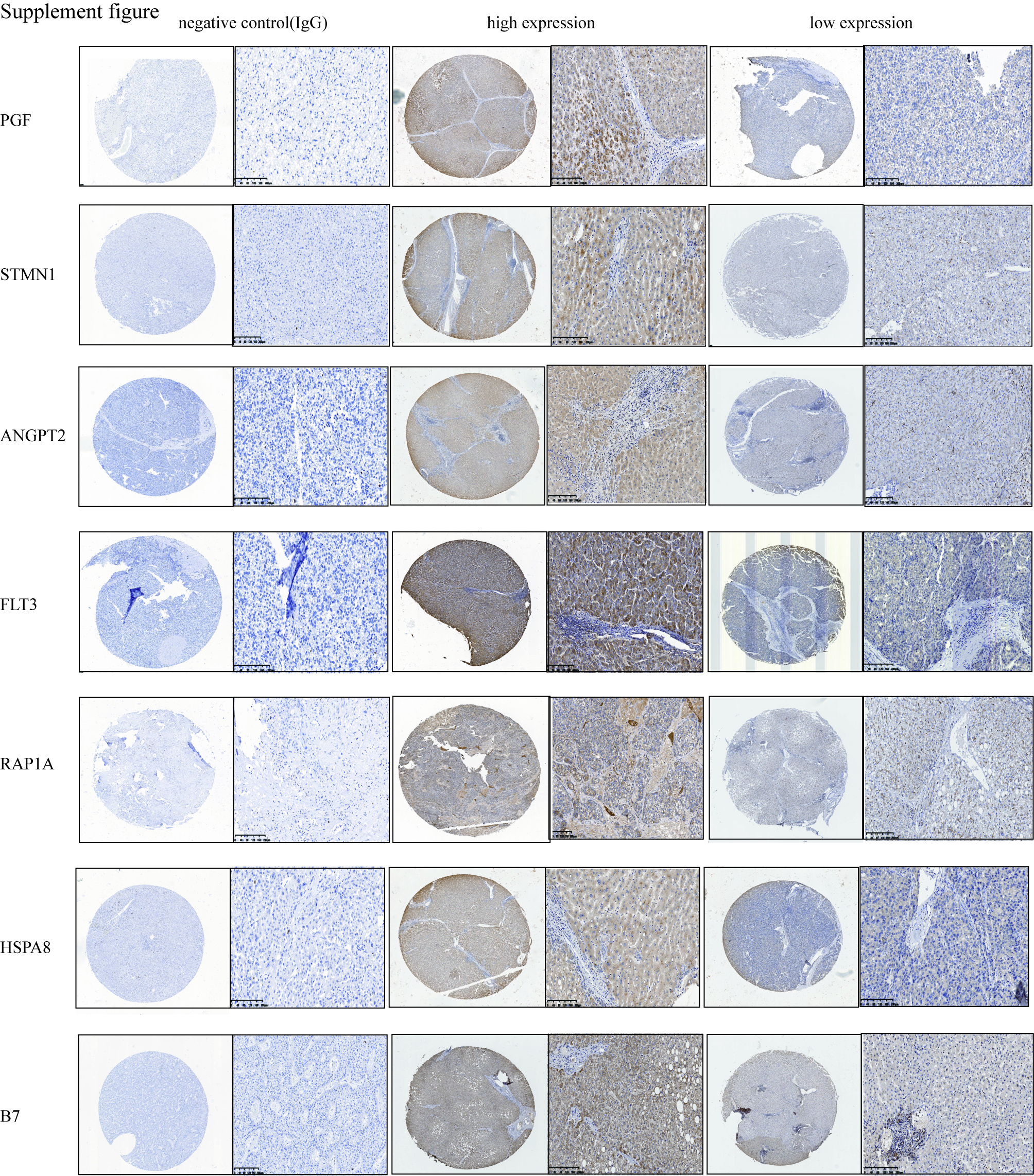

Supplement: Supplementary file 1 [file Image_1.tif]
